# Single cell transcriptomic analysis reveals pathogenic cell heterogeneity and candidate inflammatory-associated markers in STZ-induced diabetic mouse retina

**DOI:** 10.3389/fimmu.2026.1827122

**Published:** 2026-04-29

**Authors:** Shuai Ouyang, Jingwen Wang, Xiaolan Du, Shouyue Zhang, Shijun Han, Xiaotong Xu, Beichen Ren, Weihong Yu

**Affiliations:** 1 Department of Ophthalmology, Peking Union Medical College Hospital, Chinese Academy of Medical Sciences and Peking Union Medical College, Beijing, China; 2 Research Unit of Myopia Basic Research and Clinical Prevention and Control, Chinese Academy of Medical Sciences (2019RU025), Wenzhou, Zhejiang, China; 3 Beijing Key Laboratory of Fundus Diseases Intelligent Diagnosis & Drug/Device Development and Translation, Beijing, China; 4 Key Laboratory of Ocular Fundus Diseases, Chinese Academy of Medical Sciences, Beijing, China; 5 Department of Occupational Health and Environmental Health, School of Public Health, Anhui Medical University, Hefei, Anhui, China; 6 State Key Laboratory of Ophthalmology, Optometry and Visual Science, Eye Hospital, Wenzhou Medical University, Wenzhou, China

**Keywords:** cell heterogeneity, diabetic retinopathy, inflammation, retinal Müller glial cell, single-cell transcriptomic, transcriptional perturbation

## Abstract

**Background:**

Diabetic retinopathy (DR) is driven by chronic hyperglycemia and involves coordinated vascular, inflammatory, and neuroglial dysfunction. Müller glia are central to retinal homeostasis, yet their cell-state heterogeneity and inflammatory response programs in DR mice remain incompletely characterized at single-cell resolution.

**Methods:**

We reanalyzed a public scRNA-seq dataset of STZ-induced diabetic mouse retinas to characterize retinal cell populations and Müller glial states through clustering, perturbation, trajectory, functional enrichment, and co-expression network analyses, with selected targets further validated by Western blot.

**Results:**

Integration and clustering of the scRNA-seq dataset identified the major retinal cell types as well as four distinct Müller glial subpopulations. Among annotated retinal cell populations, Müller glia showed the strongest transcriptional perturbation in the STZ group, indicating that they are among the most transcriptionally responsive retinal cell types under diabetic stress. Pseudotime analysis supported the presence of branch-dependent transcriptional programs among Müller subclusters and suggested that STZ conditions were associated with preferential progression toward a Müller substate enriched for photoreceptor-associated transcripts. Functional enrichment analysis showed that different Müller glial subclusters were associated with distinct biological processes, while sharing activation of inflammatory-response programs, and highlighted *Cebpb* as a candidate inflammation-associated factor. Co-expression network analysis further identified Müller glia-associated gene modules with subcluster- and condition-dependent activity patterns, including modules linked to photoreceptor-associated programs, stress responses, angiogenesis and inflammatory signaling. Protein-protein interaction analysis prioritized *Junb* as a highly connected candidate regulatory hub, and western blotting provided supportive tissue-level evidence for altered JUNB and CEBPB protein abundance in STZ versus control retinas.

**Conclusion:**

We delineated the transcriptional heterogeneity of Müller glia and identified candidate state-associated modules and regulators linked to diabetic retinal stress responses. These findings support an active role for Müller glia in diabetic retinal remodeling through inflammatory, structural, and neuron-interactive programs, and provide a basis for future mechanistic and translational investigation of Müller glia-mediated pathology in diabetic retinopathy.

## 1 Introduction

Diabetic retinopathy (DR) is a leading cause of blindness among working-aged individuals, and its development is closely associated with progressive damage to the retinal vasculature and neuronal structures caused by prolonged hyperglycemia (1). The pathophysiology of DR involves complex molecular processes, including endothelial cell dysfunction, neuroinflammation, and disruption of retinal homeostasis (2). Although our understanding of the cellular and molecular mechanisms underlying DR has advanced, effective therapeutic strategies remain limited.

Previous studies have established the role of the retinal vasculature in DR, with some research suggesting that neurons have traditionally been regarded mainly as downstream victims of diabetic injury (3). DR is not solely a microvascular disease but also involves neurodegenerative and neuroinflammatory changes in the neural retina, including early neuronal dysfunction prior to overt vascular pathology (4). For example, glial, neural, and microvascular dysfunction have been shown to be interdependent in DR progression, and neurodegeneration itself is considered a critical aspect of the disease beyond purely vascular injury. However, increasing evidence now suggests that retinal glial cells, particularly Müller glia, play a crucial role as active participants in the progression of DR. Their functional alterations have been implicated in DR severity, inflammation, and retinal remodeling (5, 6). Müller glia are the primary glial cells in the retina, responsible for maintaining retinal structural and functional integrity under normal conditions, and they play a pivotal role in sustaining retinal homeostasis (7). Under diabetic stress, Müller glia undergo significant transcriptional and functional changes, with their dysfunction under hyperglycemic stress being closely linked to DR progression (8, 9). In the diabetic retina, Müller cells exhibit reactive gliosis, altered cytokine and growth factor production, disruption of glutamate metabolism, and contributions to inflammatory signaling, processes that are biologically linked to neuronal distress, vascular leakage, and progression of retinopathy pathology (10–13). However, the exact mechanisms by which Müller glia respond to hyperglycemia and how their dysfunction contributes to retinal degeneration remain poorly understood. More broadly, the retina can be understood within a central nervous system (CNS) context, in which localized molecular changes in specific cell types may influence tissue-level vulnerability and larger neurobiological outcomes. Within this framework, Müller glial transcriptional remodeling in diabetic retinopathy may represent a retina-specific instance of a general CNS principle whereby stress-responsive, cell-specific programs drive neural adaptation and pathological remodeling. This perspective provides additional rationale for resolving Müller glial heterogeneity and state-dependent inflammatory programs at single-cell resolution in the diabetic retina.

To clarify how Müller glia respond to hyperglycemia and how their dysfunction drives retinal degeneration, it is essential to first elucidate the specific transcriptional heterogeneity of Müller cells under diabetic conditions. Although previous studies have reported transcriptional alterations in Müller glia in diabetic retinopathy across species (14–16), a systematic characterization of Müller glial state heterogeneity and diabetes-associated transcriptional programs in the STZ-induced mouse retina remains lacking. Recent advances in single-cell RNA sequencing (scRNA-seq) technology provide an opportunity to resolve these state-specific changes at single-cell resolution and to prioritize candidate regulators relevant to diabetic retinal remodeling. Although scRNA-seq studies of diabetic retinopathy have emerged, detailed molecular profiling and analyses of specific neuronal cell types, particularly Müller glia, remain relatively limited. Single-cell transcriptomic data from STZ-induced diabetic mouse retinas offer an opportunity to explore the molecular characteristics of Müller glia in diabetic retinopathy. Leveraging single-cell transcriptomic datasets enables the identification of transcriptional perturbations associated with Müller glial dysfunction, facilitates a refined dissection of Müller glial heterogeneity, and supports the discovery of candidate biomarkers.

In this study, we systematically reanalyzed single-cell transcriptomic data from STZ-induced diabetic mouse retinas to characterize the transcriptional heterogeneity of Müller glia and identify state-associated programs linked to diabetic stress. We further sought to prioritize candidate regulators, pathways, and markers that may contribute to Müller glia–mediated retinal remodeling in diabetic retinopathy. By doing so, this study provides a transcriptomic framework for future mechanistic and translational investigations of Müller glial dysfunction in DR.

## 2 Materials and methods

### 2.1 Single-cell data acquisition and preprocessing

Public scRNA-seq data were obtained from the Gene Expression Omnibus (GEO) under accession number GSE178121 (17). In the original study, retinal tissues were collected 25 weeks after streptozotocin (STZ) induction from pooled control and diabetic mice (three mice per group). Analyses were performed in R (v4.4.2) using Seurat (v4.4.0) (18). Putative doublets were removed using DoubletFinder (v2.0.3) (19). Cells with fewer than 200 detected genes, more than 3,000 detected genes, or mitochondrial transcript proportion >20% were excluded. Low-abundance genes were also removed. After quality control, UMI counts were normalized and scaled, and the top 2,000 highly variable genes were selected. Datasets were integrated using Seurat’s canonical correlation analysis workflow. Principal component analysis was performed on the integrated matrix, and the top 30 principal components were used for shared nearest-neighbor graph construction and UMAP visualization. Cells were clustered using graph-based clustering and annotated according to established retinal cell-type marker genes.

### 2.2 Single-cell transcriptional perturbation analysis

Cell-type-specific transcriptional perturbation was quantified using Augur (v1.0.3) (20). The normalized expression matrix, cell-type annotations, and condition labels (STZ vs. control) were used as input. For each cell type, Augur generated balanced subsamples, trained a classifier to distinguish conditions, and summarized performance using the area under the ROC curve. Classifier performance was summarized by the area under the receiver operating characteristic curve (AUC) (21, 22). Cell types were ranked by mean AUC, with higher values indicating stronger transcriptional perturbation.

### 2.3 State-transition potential estimation and pseudotime trajectory inference

CytoTRACE (v0.3.3) and Monocle2 (v2.26.0) were used to estimate relative transcriptional plasticity and infer pseudotime trajectories (23, 24). CytoTRACE was applied to the normalized expression matrix to calculate a score for each cell, with higher scores interpreted as relatively greater transcriptional plasticity. For pseudotime analysis, Seurat-derived expression data and metadata were converted into a Monocle2 CellDataSet. Ordering genes were selected from genes with substantial variation across the dataset, and dimensionality reduction was performed using DDRTree. Cells were ordered along pseudotime and assigned to branch states. The trajectory root was defined as the state enriched for cells with the highest CytoTRACE scores. Branch-dependent gene-expression dynamics were visualized using plot_genes_branched_pseudotime().

### 2.4 Differential gene expression analysis

Differential expression analysis was performed using MAST (v1.32.0) as implemented in Seurat (25). Differential expression analysis was performed on log-normalized expression data while adjusting for cellular detection rate and library size as covariates. Genes with an absolute log2 fold change ≥0.25 and Benjamini-Hochberg-adjusted p < 0.05 were considered differentially expressed.

### 2.5 Functional enrichment analyses

Functional enrichment was assessed using Metascape and pre-ranked gene set enrichment analysis (GSEA) implemented in clusterProfiler (v4.19.1) (26, 27). For over-representation analysis, genes from each module or selected DEG set were submitted to Metascape using Gene Ontology Biological Process terms, and terms with p < 0.05 were considered significant. For GSEA, genes were ranked by log2 fold change and analyzed using gseGO() with OrgDb = org.Mm.eg.db, ont = “BP”, minGSSize = 10, maxGSSize = 500, and pvalueCutoff = 0.05. Gene sets with NES > 0 and p < 0.05 were considered positively enriched, whereas those with NES < 0 and p < 0.05 were considered negatively enriched.

### 2.6 Protein–protein interaction network construction and hub gene identification

Protein-protein interaction (PPI) networks were constructed using STRING (version 12.0) (https://string-db.org) (28). Gene sets were submitted using the “multiple proteins” option, with interaction type set to “evidence” and a minimum interaction score of 0.400. Networks were imported into Cytoscape (v3.9.0) for visualization and analysis (29). Significant subnetworks were identified using MCODE (30) with default parameters, and hub genes were ranked using cytoHubba based on Maximal Clique Centrality (MCC) (31).

### 2.7 Gene co-expression network analysis

High-dimensional weighted gene co-expression network analysis (hdWGCNA) was performed using hdWGCNA (v0.4.0) in R (32) using the integrated Seurat object containing Müller glia. The object was prepared using SetupForWGCNA(). Candidate soft-thresholding powers were evaluated using TestSoftPowers(), and β = 5 was selected as the minimal power at which the scale-free topology fit index exceeded 0.8 while maintaining adequate network connectivity. Co-expression networks were constructed using ConstructNetwork(), and module eigengenes were derived for each module. Hub genes were visualized using PlotKMEs(), and module-level activation scores were calculated using ModuleExprScore().

### 2.8 Establishment of the STZ-induced diabetic retinopathy mouse model and retinal tissue isolation

All animal experiments were approved by the Ethics Committee of Peking Union Medical College Hospital (No. XHDW-2025-032). Six-week-old C57BL/6J male mice were fasted overnight and injected intraperitoneally with streptozotocin (STZ, 55 mg/kg) dissolved in citrate buffer (pH 4.5). Control mice received citrate buffer alone. Mice with fasting blood glucose >300 mg/dL were considered diabetic. After 8 weeks, mice were euthanized with sodium pentobarbital (100 mg/kg, intraperitoneally) followed by cervical dislocation. Eyes were enucleated, and retinas were isolated and stored at −80 °C for protein analysis.

### 2.9 Western blot validation of candidate protein expression

Retinal tissues were lysed in RIPA buffer (Beyotime, P0038) containing protease and phosphatase inhibitors. After homogenization and centrifugation, supernatants were collected and protein concentration was determined using a BCA assay (Beyotime, P0009). Equal amounts of protein were separated by SDS-PAGE and transferred to PVDF membranes. Membranes were blocked with 5% nonfat milk in TBST for 1 h at room temperature and incubated overnight at 4 °C with primary antibodies against JUNB (Abmart, T55894, 1:2000), CEBPB (Abmart, T55276, 1:2000), and β-actin (Affinity, AF7018, 1:20000). After washing, membranes were incubated for 1 h at room temperature with HRP-conjugated secondary antibodies. Protein bands were visualized using enhanced chemiluminescence (ECL).

### 2.10 Statistical analysis

Western blot quantification data were analyzed using GraphPad Prism (v10.3.1). Comparisons between STZ and control groups were performed using unpaired two-tailed t-tests. Single-cell analyses and visualization were performed in R (v4.4.2). Statistical significance was defined as p < 0.05. Statistical significance in the figures is indicated as: p < 0.05 (*), p < 0.01 (**), p < 0.001 (***), and p < 0.0001 (****).

## 3 Results

### 3.1 Construction of an STZ-induced mouse DR single-cell transcriptomic atlas identifies Müller glia as the most transcriptionally perturbed population

**Figure 1:**
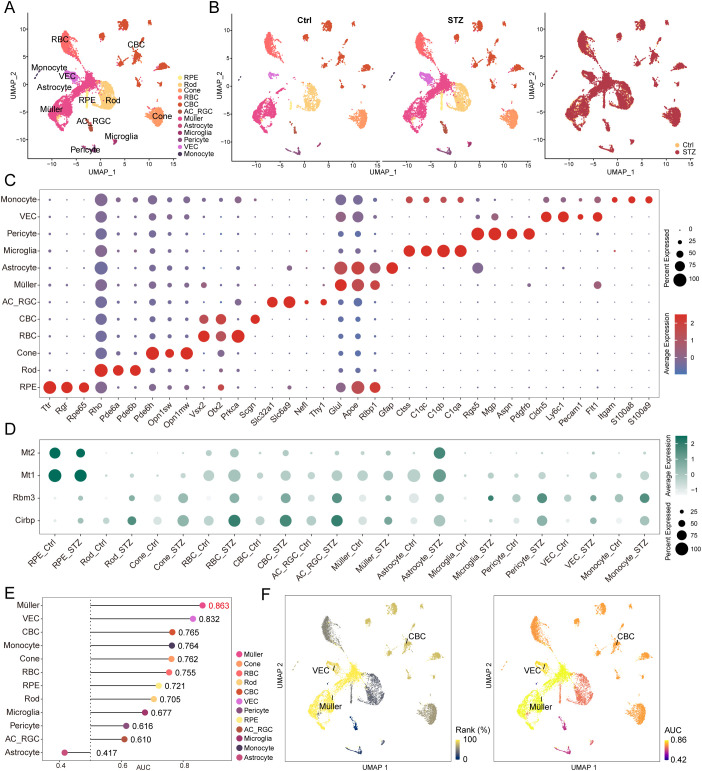
Single-cell annotation of retina from STZ-induced diabetic retinopathy mice. **(A)** UMAP visualization of cell type annotations within the retina from control and STZ-induced diabetic mice. **(B)** UMAP projection showing the distribution of different cell types across both the Ctrl and STZ groups, with no apparent batch effect following data integration. **(C)** Bubble plot depicting the expression levels of cell-type specific marker genes across different retinal cell populations. **(D)** Bubble plot illustrating widely upregulated genes in various retinal cell types in the STZ group. **(E)** Lollipop plot displaying transcriptional perturbation scores for different retinal cell types in the STZ group, with Müller cells exhibiting the most pronounced perturbation. **(F)** UMAP plot showing the top three cell types with the highest transcriptional perturbation, ranked by AUC and perturbation scores.

We retrieved and analyzed the public single-cell RNA-sequencing dataset GSE178121, which profiles retinal transcriptomes from healthy mice and streptozotocin (STZ)-induced diabetic mice. Following stringent quality control, batch-effect correction, and standard preprocessing, a total of 14,175 cells were retained, including 5,325 cells from the control group and 8,850 cells from the STZ group (**Figure 1A**). Major retinal cell populations were annotated using well-established, cell type–specific marker genes. These populations included retinal pigment epithelium (RPE; *Ttr, Rgr, Rpe65*), rods (*Rho, Pde6a, Pde6b*), cones (*Pde6h, Opn1sw, Opn1mw*), rod bipolar cells (RBC; *Vsx2, Otx2, Prkca*), cone bipolar cells (CBC; *Vsx2, Otx2, Prkca*), amacrine cells and retinal ganglion cells (AC/RGC; *Slc32a1, Slc6a9, Nefl, Thy1*), Müller glia (*Glul, Apoe, Rlbp1*), astrocytes (*Apoe, Gfap*), microglia (*Ctss, C1qa, C1qb, C1qc*), pericytes (*Rgs5, Mgp, Aspn, Pdgfrb*), vascular endothelial cells (VECs; C*ldn5, Ly6c1, Pecam1, Flt1*), and monocytes (*Itgam, S100a8, S100a9*) (**Figures 1B, C**). Visualization of cell-type composition across experimental groups and batches indicated that all major populations were captured in both conditions, and no obvious batch-driven separation was observed between the control and STZ samples (**Figure 1B**). A bubble plot further confirmed that canonical markers were strongly enriched within their corresponding clusters, supporting accurate annotation (**Figure 1C**, **Supplementary Figure 1A**).

To further evaluate the robustness of clustering and annotation, we assessed the expression of representative stress-responsive genes, including the cold-inducible RNA-binding proteins (*Cirbp* and *Rbm3*) and the metallothioneins (*Mt1* and *Mt2*). As expected in the context of an STZ-induced diabetic stress context, these transcripts were broadly upregulated across multiple retinal cell types in the STZ group relative to controls (**Figure 1D**), consistent with prior observations in experimental diabetes and supporting the reliability of our analytical workflow (17).

Next, to identify the retinal cell populations most responsive to STZ-induced diabetes at the transcriptomic level, we quantified cell-type–specific perturbation using the Augur framework. Among all annotated populations, Müller glia displayed the highest perturbation score (AUC = 0.863), followed by vascular endothelial cells (AUC = 0.832) and cone bipolar cells (AUC = 0.765) (**Figure 1E**). These findings indicate that Müller glia undergo the most pronounced transcriptional reprogramming in STZ-induced DR, highlighting Müller glia as a major cell population involved in diabetes-associated molecular remodeling. We further projected rank-based and AUC-based perturbation scores onto the UMAP embedding to visualize the spatial distribution of the three most perturbed populations (**Figure 1F**).

In summary, we constructed a single-cell transcriptomic atlas of the STZ-induced diabetic mouse retina, validated cell-type annotation using canonical markers and diabetes-associated stress signatures, and identified Müller glia as the population exhibiting the strongest transcriptional perturbation. This result highlights Müller glia as a key cellular compartment for investigating diabetes-driven retinal remodeling and DR-related pathogenic mechanisms.

### 3.2 Pronounced heterogeneity of Müller glial subpopulations and a potential transcriptional state transition program

**Figure 2:**
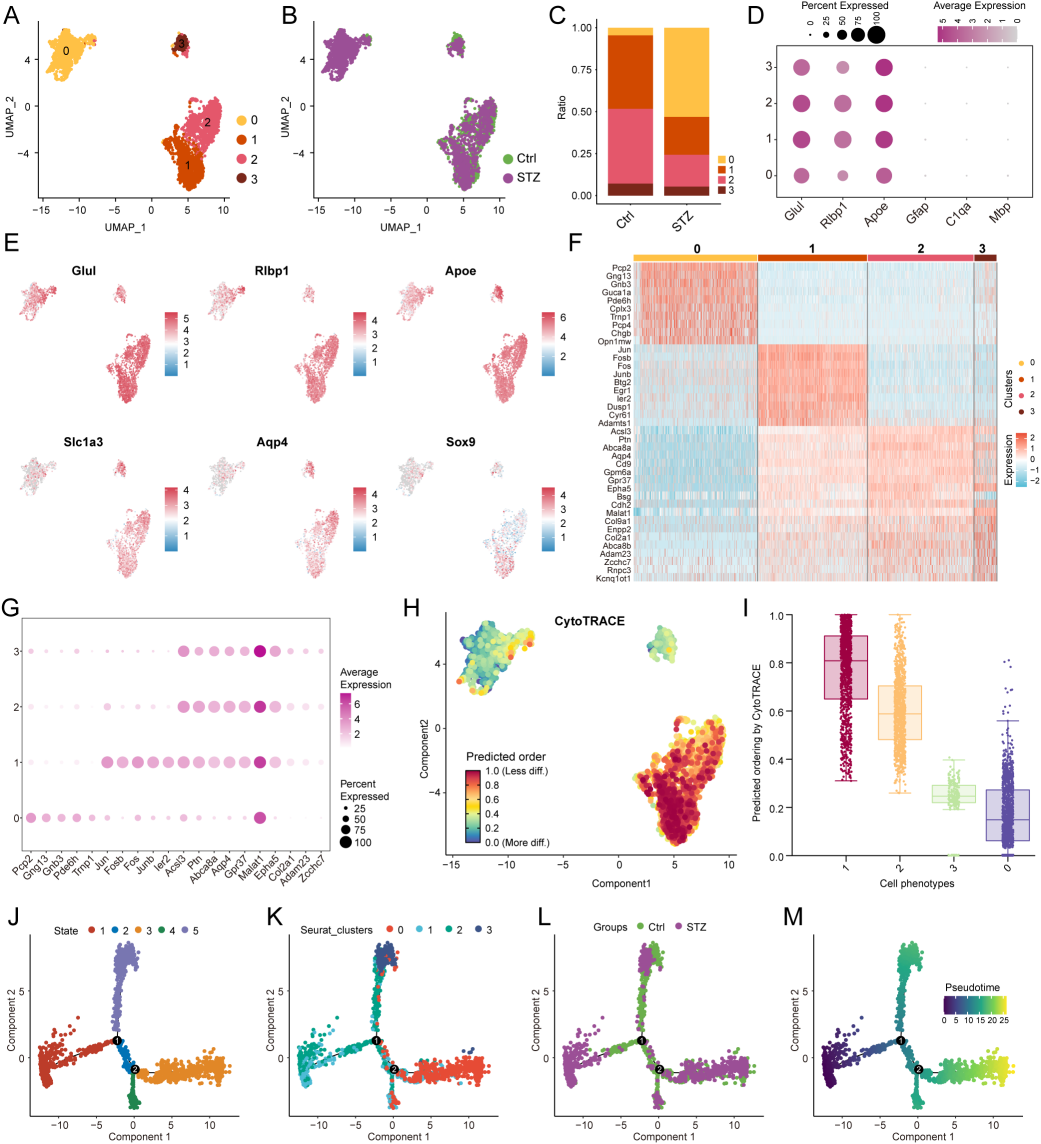
Müller cell subpopulation analysis and pseudotime state-transition trajectories. **(A)** UMAP showing the clustering of Müller cell subpopulations in the retina. **(B)** UMAP highlighting the absence of significant batch effects between the control and STZ groups after integration. **(C)** Bar plot depicting the relative proportions of different Müller cell subpopulations under control and STZ conditions. **(D)** The dot plot showing the expression levels of marker genes for Müller glia (*Glul, Rlbp1, Apoe*), astrocytes (*Gfap*), microglia (*C1qa*), and oligodendrocytes (*Mbp*) across the four Müller cell subclusters. **(E)** Feature plot showing the expression patterns of Müller cell markers (*Glul, Rlbp1, Apoe, Slc1a3, Aqp4, Sox9*) across the various subpopulations. **(F)** Heatmap displaying the most highly expressed genes in each Müller cell subpopulation. **(G)** Dot plot illustrating the top-expressed genes in each Müller cell subpopulation. **(H)** CytoTRACE analysis revealing the relative state-transition potential of each Müller subpopulation. **(I)** Box plot showing the relative state-transition potential (CytoTRACE scores) for each Müller subpopulation. **(J–M)** Monocle 2 pseudotime analysis revealing potential reactive state-transition trajectories among Müller cell subpopulations, presented across different states, clusters, groups, and pseudotime.

Given that Müller glia exhibited the strongest transcriptional perturbation in STZ-induced DR retinas, we next focused on Müller cells to further resolve their intrapopulation heterogeneity. We subsetted Müller glia and re-integrated the data with additional batch correction, which resulted in four transcriptionally distinct subclusters (Clusters 0–3) (**Figures 2A, B**). We then quantified the relative abundance of each subcluster across conditions and observed a trend toward an increased relative proportion of Cluster 0 in the STZ group (**Figure 2C**). This compositional pattern suggests that STZ-induced diabetes may be associated with remodeling of Müller glial states, potentially reflecting functional diversification and/or preferential reinforcement of specific cell states. To confirm that all identified subclusters indeed represented Müller glia, we examined canonical Müller markers using a dot plot and UMAP feature maps, including *Glul, Rlbp1, Apoe, Slc1a3, Aqp4*, and *Sox9*. Classical Müller glial markers such as *Glul*, *Rlbp1*, and *Apoe* were broadly expressed across all four subclusters whereas marker genes for other glial cell types were barely detectable, supporting the validity of our Müller-specific subclustering (**Figures 2D, E**). We next characterized transcriptional differences among Müller subclusters by identifying highly expressed genes in each cluster and visualizing these signatures using a heatmap and dot plot (**Figures 2F, G**). Notably, Cluster 0 was enriched for genes typically associated with photoreceptor or bipolar cell identity, including *Pcp2, Gng13, Pde6h*, and *Opn1mw*. Cluster 1 displayed elevated expression of immediate-early and stress/inflammatory response genes, such as *Jun, Fos, Fosb*, and *Junb*. Cluster 2 preferentially expressed genes related to lipid metabolism and transport, including *Acsl3, Ptn*, and *Abca8a*. Cluster 3 showed higher expression of transcripts involved in RNA regulation and extracellular matrix (ECM) programs, including *Malat1, Zcchc7, Col9a1*, and *Col2a1* (**Figures 2F, G**). Collectively, these findings indicate pronounced transcriptional heterogeneity within Müller glia and suggest that each subcluster may represent a distinct functional state.

To explore whether these subclusters differed in relative transcriptional plasticity, we applied CytoTRACE to infer state-transition potential across Müller states. Clusters 1 and 2 exhibited the highest inferred state-transition potential, whereas Clusters 0 and 3 showed comparatively lower scores (**Figures 2H, I**). This pattern is consistent with a model in which Clusters 0 and 3 may represent relatively more specialized reactive states that are transcriptionally downstream of the higher-plasticity states represented by Clusters 1 and 2. We further reconstructed a putative reactive state-transition trajectory using Monocle 2 pseudotime analysis. Along the inferred trajectory, Clusters 1 and 2 were positioned near the pseudotime origin, whereas Cluster 0 was predominantly located toward the later portion of the trajectory (**Figures 2J–M**). These results support the presence of a potential transcriptional progression among Müller subclusters, in which higher-plasticity states represented by Clusters 1 and 2 are associated with downstream reactive states, including Clusters 3 and 0. Importantly, the trend toward increased representation of Cluster 0 in STZ-induced DR suggests that the trajectory leading to this late reactive state is preferentially reinforced under diabetic stress.

**Figure 3:**
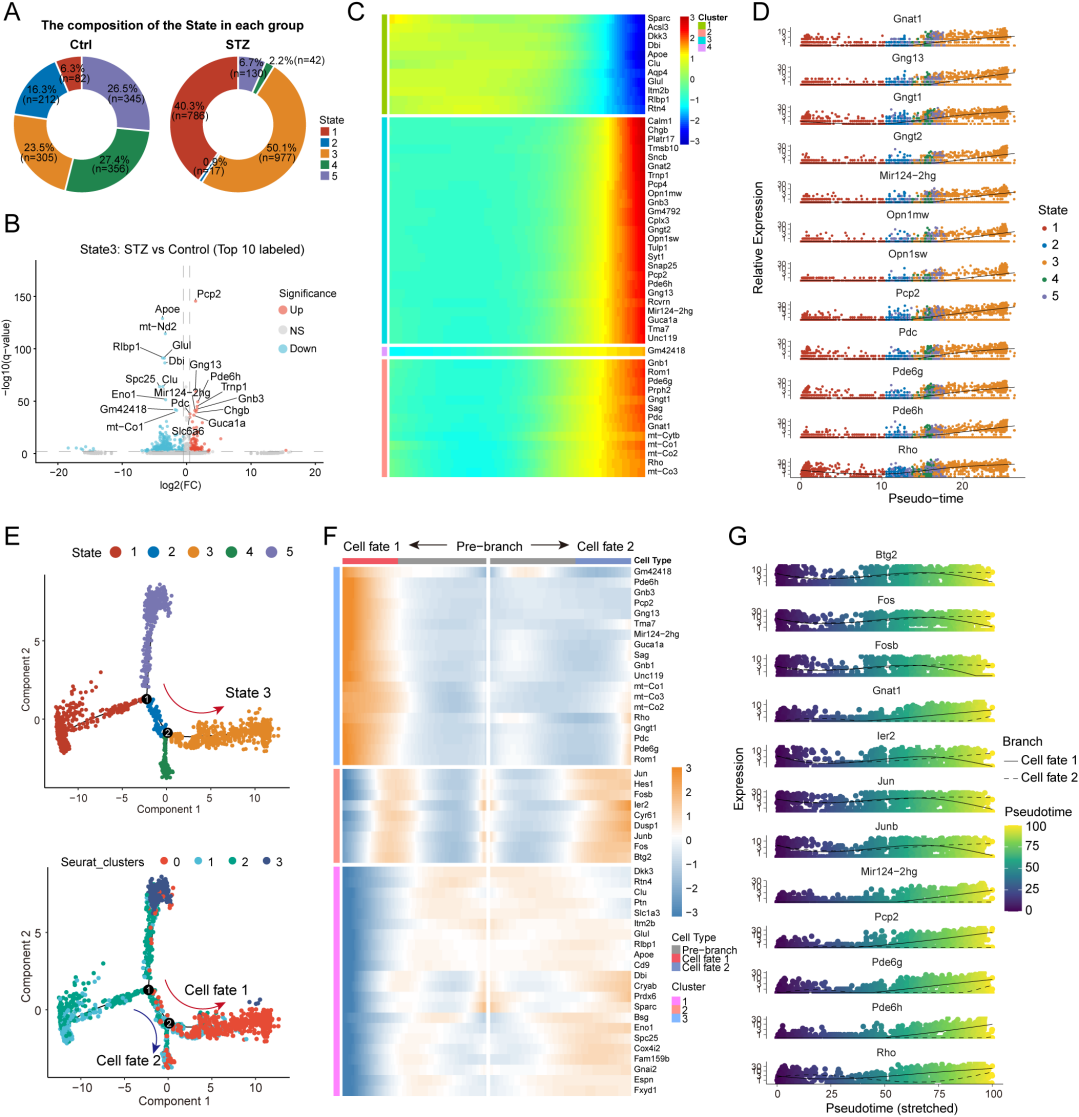
STZ-induced enhancement of specific Müller cell state-transition trajectories. **(A)** Circle plot showing the proportion of cells within different pseudotime states across the control and STZ groups. **(B)** Volcano plot showing differentially expressed genes in State 3 in STZ-treated cells compared to control, highlighting upregulated and downregulated genes. **(C)** Heatmap illustrating temporal changes in gene expression as cells progress along the pseudotime trajectory toward State 3. **(D)** Gene trajectory plot showing the expression patterns of genes that progressively increase as cells approach State 3. **(E)** Pseudotime trajectory analysis showing progression from State 1 to State 3, including bifurcation into cell fate 1 and cell fate 2 at the second inflection point. **(F)** Heatmap displaying gene expression changes as cells progress from the pre-branch region to cell fate 1 and cell fate 2. **(G)** Gene trajectory plot depicting the expression trends of specific genes along the differentiation paths toward cell fate 1 and cell fate 2.

To directly evaluate branch usage across conditions, we compared the proportion of cells distributed along distinct Monocle-defined branches between the Ctrl and STZ groups. This analysis revealed a higher relative proportion of cells within the State 3 branch under STZ conditions (**Figure 3A**). Consistent with the pseudotime mapping, cells occupying State 3 was composed predominantly of Cluster 0 cells, further supporting enhanced transition toward the Cluster 0 reactive state in diabetic retinas. We next performed differential expression analysis within State 3 to identify genes altered under STZ relative to control. Genes upregulated in STZ-enriched State 3 included *Pcp2, Gng13*, and *Pde6h*, which are commonly associated with photoreceptors or bipolar cells, whereas genes downregulated included Müller-associated transcripts such as *Apoe, Clu*, and *Rlbp1* (**Figure 3B**). These changes suggest that, under STZ-induced stress, a subset of Müller glia may partially lose canonical Müller identity features and acquire expression patterns consistent with increased interaction with, or clearance of, damaged neuronal elements.

We next visualized the expression of these genes within Cluster 0 under STZ and Ctrl conditions using a bubble plot. Consistent with our enrichment results, photoreceptor-associated marker genes exhibited higher expression in Cluster 0 under STZ compared with controls (**Supplementary Figure 1B**). To delineate dynamic gene expression changes along the trajectory toward State 3, we visualized pseudotime-dependent expression patterns using a trajectory heatmap (**Figure 3C**). We further examined the expression of photoreceptor-related marker genes along pseudotime and observed progressive upregulation of *Pde6g, Pde6h, Rho, Opn1mw, Opn1sw, Pcp2*, and *Gng13* as cells advanced toward State 3 (**Figure 3D**). Because State 3 corresponds to the later region of the inferred trajectory, these findings are consistent with the notion that this state is transcriptionally downstream of earlier Müller reactive states (**Figures 2M**, **3E**).

Notably, the pseudotime trajectory contained two inflection points. We defined the segment prior to the second inflection as the pre-branch region and the two divergent post-inflection directions as cell fate 1 and cell fate 2. Cluster 0 cells were primarily located after the second inflection, indicating that Cluster 0 is preferentially associated with the branch corresponding to the cell fate 1 path. This observation supports the existence of structured reactive state transitions within Müller glia and suggests that multiple transcriptional routes may enable Müller cells to adopt distinct stress-associated functional states. We then characterized branch-specific transcriptional programs by comparing gene dynamics between the two fate directions. Genes enriched along cell fate 1 were dominated by photoreceptor-associated markers such as *Rho*, *Pde6g*, and *Pde6h*. In contrast, genes enriched along cell fate 2 were primarily associated with stress and inflammatory signaling, including *Jun, Junb, Fos, Fosb, Ier2*, and *Cyr61* (**Figure 3F**). We further visualized representative gene expression trajectories across pseudotime and fate directions (**Figure 3G**). Finally, we noted that the long non-coding RNA *Mir124-2hg* was highly expressed in State 3 and displayed a progressive increase along the cell fate 1 direction (**Figures 3D,G**). This observation raises the possibility that *Mir124-2hg* may be associated with STZ-associated Müller glial state transitions and merits further mechanistic investigation.

### 3.3 STZ induces transcriptional remodeling of Müller glial substates and activates inflammatory programs

**Figure 4:**
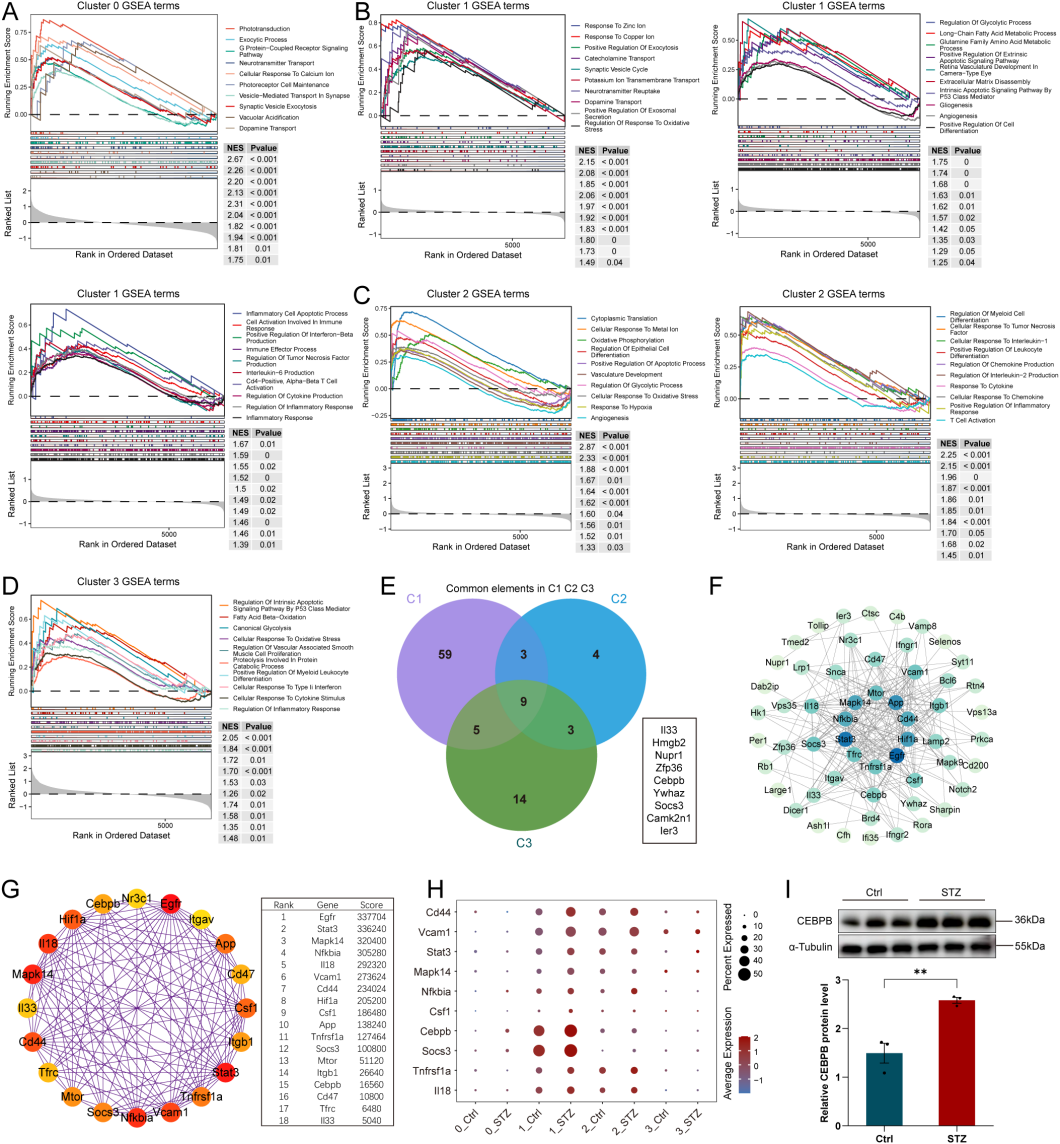
Biological pathways activated in Müller subpopulations under STZ conditions. **(A)** Gene set enrichment analysis (GSEA) results showing the activated biological pathways in Müller cells of Cluster 0. **(B)** GSEA results revealing the activated pathways in Müller cells of Cluster 1. **(C)** GSEA results indicating the functional pathways activated in Müller cells of Cluster 2. **(D)** GSEA results demonstrating the functional pathways activated in Müller cells of Cluster 3. **(E)** Intersection of genes enriched in the inflammatory response pathways across Clusters 1, 2, and 3. **(F)** Protein-protein interaction (PPI) network analysis of genes enriched in inflammatory pathways in Cluster 1. **(G)** Top 20 hub genes identified from the PPI network in **(F)** based on connectivity scores. **(H)** Bubble plot showing expression level changes of the top 20 hub genes in Müller subpopulations across different conditions, with a focus on inflammation-related genes. **(I)** Western blot validation of CEBPB protein expression levels in the Ctrl and STZ groups (N = 3), confirming upregulation in STZ-treated mice. ** indicates p < 1.

To further delineate STZ-associated transcriptional heterogeneity among Müller glial substates, we performed functional enrichment analyses for each Müller subcluster. Under STZ exposure, Cluster 0 showed prominent upregulation of pathways related to phototransduction, endocytosis, neurotransmitter transport, photoreceptor maintenance, synaptic pruning/phagocytosis, and dopamine transport. These enrichments suggest that diabetic stress may be accompanied by compensatory programs aimed at sustaining photoreceptor-related function, together with an enhanced phagocytic capacity of Müller glia in the context of neuronal injury (**Figure 4A**). In contrast, Cluster 1 exhibited broad activation across diverse biological processes, including responses to ions, endocytosis and vesicle trafficking, glycolytic metabolism and fatty acid metabolism, apoptosis, extracellular matrix disassembly, gliosis, angiogenesis, and cellular differentiation. The enrichment of differentiation-related programs is consistent with our pseudotime analyses, which indicated that Clusters 1 and 2 occupy earlier positions along trajectories that progress toward the Cluster 0 branch. Notably, Cluster 1 also displayed strong activation of immune and inflammatory pathways, including immune activation-associated responses, interferon signaling, interleukin-6 production, T-cell activation, and generalized inflammatory response programs (**Figure 4B**). Cluster 2 was enriched for translational processes and oxidative phosphorylation, together with pathways related to cellular differentiation, vascular development, glycolysis, hypoxia, angiogenesis, cytokine production, and inflammatory responses (**Figure 4C**). Cluster 3 preferentially activated pathways linked to apoptosis, fatty acid oxidation, glycolysis, cytokine stimulus responses, and inflammatory signaling (**Figure 4D**). Importantly, inflammatory-response programs were consistently enriched across Clusters 1, 2, and 3, indicating that multiple Müller substates engage inflammatory modules under STZ-induced diabetic stress.

To identify shared inflammatory-response drivers across these substates, we intersected the gene sets contributing to the inflammatory-response enrichments in Clusters 1, 2, and 3. This analysis yielded nine common genes associated with inflammatory activation: *Il33, Hmgb2, Nupr1, Zfp36, Cebpb, Ywhaz, Socs3, Camk2n1*, and *Ier3* (**Figure 4E**). Among all subclusters, Cluster 1 contained the largest number of inflammatory-response–associated genes, suggesting that it may represent a major inflammation-linked Müller substate in STZ-induced DR. Given the prominence of Cluster 1 inflammatory signaling, we next constructed a protein–protein interaction (PPI) network using Cluster 1 genes enriched in inflammatory pathways to identify core interaction frameworks (**Figure 4F**). Hub-gene prioritization using the CytoHubba algorithm identified the top 20 hub genes and their corresponding scores (**Figure 4G**). Among these, *Il33* is of particular interest, as previous studies have demonstrated increased IL-33 expression in Müller glia in diabetic retinas with immunomodulatory functions. In addition to Il33, our hub set contained multiple factors implicated in the initiation, maintenance, or regulation of inflammation, including *Il18, Tnfrsf1a, Socs3, Cebpb, Csf1, Nfkbia, Mapk14, Stat3, Vcam1*, and *Cd44*. We further visualized the expression of these candidates across Müller subclusters and conditions using dot plots, which revealed comparatively higher expression of *Socs3* and *Cebpb* within Cluster 1 (**Figure 4H**).

Interestingly, prior studies suggest that SOCS3 may exhibit context-dependent behavior in diabetic retinal inflammation. Retinal SOCS3 dysregulation has been linked to experimental diabetes and retinal insulin resistance, whereas SOCS3 induction has also been reported to suppress inflammatory signaling in retinal immune cells (33, 34). In this context, the elevated *Socs3* expression observed in our single-cell analysis may reflect substate-specific regulation within the inflammatory Cluster 1 Müller population that is not readily captured by whole-retina measurements. Given that Müller glia account for only a small fraction of retinal cells (approximately 3%–6%), whole-retina measurements may not fully capture substate-specific regulation. We therefore selected *Cebpb*, which displayed a pronounced increase in Cluster 1 under STZ conditions, for validation at the whole-retina protein level. Western blot analysis confirmed that CEBPB protein abundance was increased (**Figure 4I**), consistent with the direction of change observed at the mRNA level.

Collectively, these results demonstrate that Müller glia comprise transcriptionally distinct substates with divergent functional programs, and that STZ-induced diabetic stress reshapes this heterogeneity. The observed substate-specific changes include putatively compensatory adaptations, such as photoreceptor-supportive and phagocytosis-associated programs that may help buffer diabetes-related neuronal injury, as well as inflammatory regulatory and maintenance programs that may contribute to retinal inflammatory remodeling in STZ-induced diabetic retinopathy.

### 3.4 HdWGCNA identifies disease-associated gene co-expression networks in retinal Müller glial substates under STZ conditions

**Figure 5:**
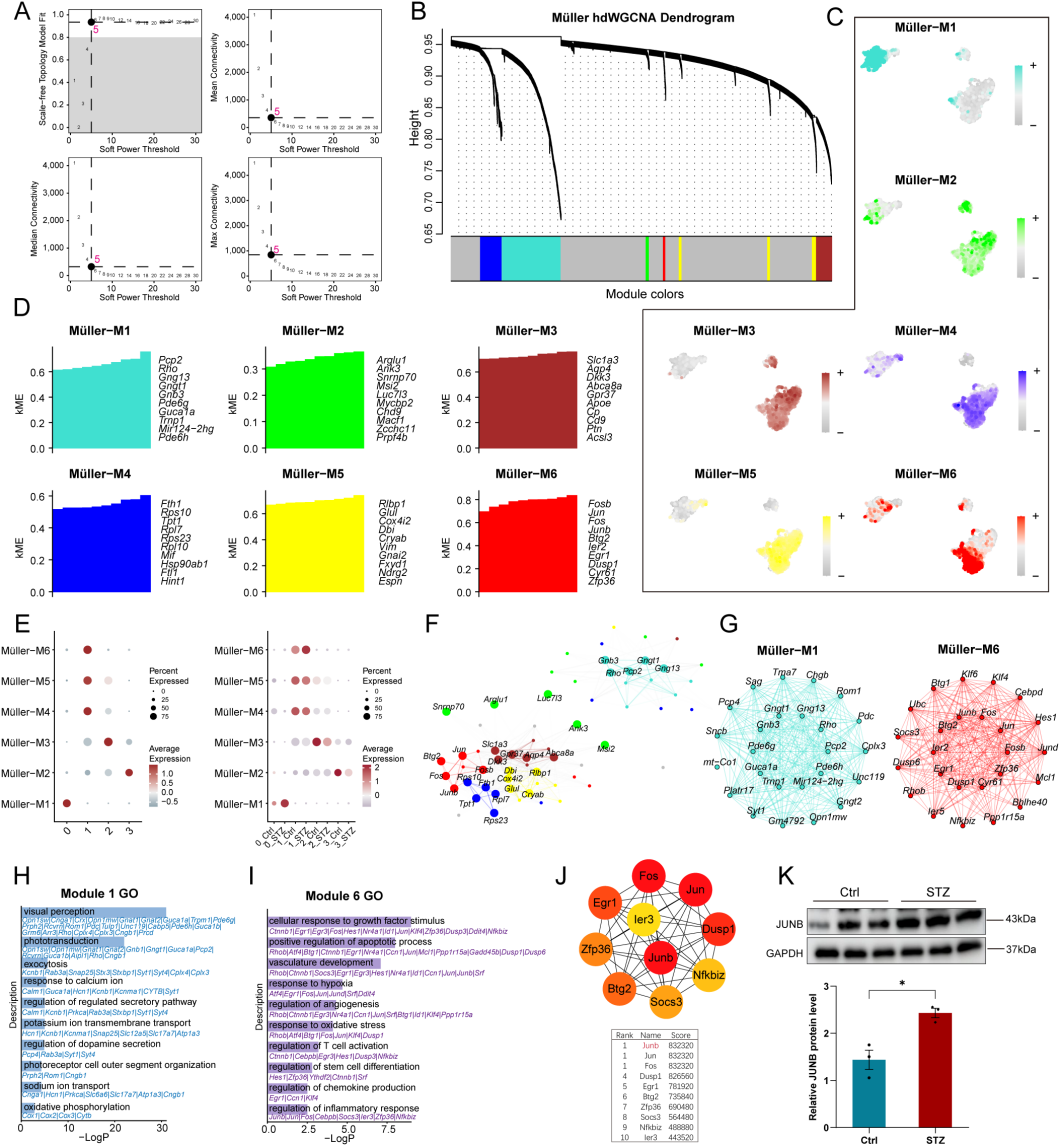
hdWGCNA analysis of gene co-expression modules and functional enrichment in Müller glial subpopulations cluster 0 and 1 in STZ and Ctrl groups. **(A)** Soft power threshold selection for the weighted network construction in the hierarchical clustering analysis of Müller glia subpopulations. The figure showed the correlation between the soft power threshold and model fit, as well as mean connectivity, median connectivity and max connectivity, with the optimal threshold indicated for constructing the network. The selected power (β = 5) represents the minimal value satisfying the scale-free topology criterion while preserving sufficient network connectivity for module detection. **(B)** Hierarchical clustering dendrogram of Müller glial subpopulations based on gene expression profiles. The tree showed clustering of the different gene modules identified in the analysis, with modules labeled Müller-M1 to Müller-M6. Each module represents a set of co-expressed genes associated with specific biological functions in Müller glial subpopulation. **(C)** Module feature plots showing the expression patterns of each module (M1-M6) within Müller cell subpopulations. For each module, cells were colored by module expression level (low to high), illustrating the distribution of module activity across Müller cell subpopulations. **(D)** The module membership (kME) for all genes was calculated, and the top-ranked 10 genes within each module were visualized using PlotKMEs. **(E)** Dot plots respectively showing the expression of distinct gene co-expression modules in Müller glial cell subpopulation and the module expression within subpopulations under different conditions. **(F)** Co-expression network comprising the top five hub genes from each co-expression module. **(G)** Network graphs showing hub-gene networks constructed from the top 25 core genes of modules 1 and 6; the inner ring denotes the top 10 hub genes and the outer ring denotes the remaining 15. **(H, I)** Bar charts showing functional enrichment terms for the top 100 genes from modules 1 and 6. **(J)** PPI network analysis of genes in Module 6, showing the top 10 hub genes selected based on connectivity scores. **(K)** Western blot validation of JUNB protein expression levels in the Ctrl and STZ groups (N = 3), confirming differential expression. * indicates p < 0.05.

To identify gene modules underlying STZ-associated remodeling of Müller glial substates and to uncover disease-relevant co-expression programs that may represent dysregulated driver networks, we performed hdWGCNA on Müller glia from STZ retinas. Using a soft-thresholding power of 5, selected as the minimal power satisfying the scale-free topology criterion while preserving adequate network connectivity, we constructed the co-expression network and identified six gene co-expression modules (**Figures 5A, B**). We next quantified and visualized module activity across all Müller subclusters by UCell-based module scoring (**Figure 5C**). In parallel, we computed kME (module membership) values for genes within each module and used PlotKMEs to display the top 10 genes ranked by kME (**Figure 5D**). To further evaluate subcluster specificity and condition dependence of module activation, we compared the expression/activity levels of the six modules across Müller subclusters and examined subcluster-wise module patterns between control and STZ conditions (**Figure 5E**). Distinct modules exhibited clear subcluster preferences. For example, Module 1 was selectively enriched in Cluster 0, whereas Module 6 was selectively enriched in Cluster 1, suggesting that these subcluster-specific modules may contribute to maintaining the core identity and functional programs of their corresponding Müller substates. Notably, under STZ conditions, Module 1 showed a trend toward increased activity in Cluster 0, and Module 6 was similarly elevated in Cluster 1, indicating that these two modules are preferentially activated within distinct Müller substates during STZ-induced DR and may contribute to STZ-associated remodeling.

To gain insight into the biological themes represented by each module, we visualized interaction networks for the top hub genes across modules. In Module 1, the top five hub genes were *Gnb3, Gngt1, Rho, Pcp2*, and *Gng1*, many of which are commonly associated with photoreceptor or bipolar cell signatures. In Module 6, the top five hub genes were *Jun, Btg2, Fos, Junb*, and *Fosb*, which are characteristic of immediate-early, stress-responsive, and inflammation-associated transcriptional programs. We further reconstructed hub-gene interaction networks for the top 25 hub genes in Modules 1 and 6. To systematically infer module function, we performed functional enrichment analyses using the top 100 hub genes from each module. Module 1 was primarily enriched for processes related to visual perception and phototransduction, endocytosis, photoreceptor outer segment organization, ion transport, and oxidative stress responses. In contrast, Module 6 was enriched for pathways associated with apoptosis, angiogenesis, responses to hypoxia, stem cell differentiation, and immune/inflammatory processes. These results indicate that Module 1 captures a photoreceptor/visual function–linked program prominent in Cluster 0, whereas Module 6 represents a stress- and inflammation-centered program preferentially active in Cluster 1. To further prioritize core regulatory genes within the Cluster 1 co-expression program, we constructed a protein–protein interaction (PPI) network from the Module 6 top 100 hub genes and applied the CytoHubba algorithm in Cytoscape to rank the top 10 PPI hub genes. *Junb* emerged as the highest-scoring node, followed by additional high-ranking factors including *Jun, Fos, Nfkbiz, Socs3, Ier3*, and *Zfp36*. Notably, these prioritized genes overlapped with inflammatory-response–associated functional terms enriched in Module 6, supporting the interpretation that inflammatory signaling constitutes a central functional axis of Module 6. Together with the observed STZ-associated increase in Module 6 activity, these findings suggest that inflammatory gene networks linked to specific Müller substates are reinforced under diabetic stress, further supporting an active role of Müller glia in shaping retinal inflammatory responses in this model.

Finally, we validated the increased activation of this inflammatory module at the protein level. Western blot analysis of whole retina confirmed that JUNB protein abundance was elevated in STZ-induced DR mice relative to controls. Collectively, these data indicate that hdWGCNA can resolve disease-associated co-expression programs in Müller glial substates and highlight a *Junb*-centered inflammatory regulatory network as a candidate target for future mechanistic and biomarker-oriented studies.

## 4 Discussion

In this study, we constructed a single-cell transcriptomic atlas of the STZ-induced diabetic retina and used a state-resolved framework to interrogate Müller glial (MG) responses to diabetic stress. Three findings stand out. First, quantitative perturbation prioritization indicated that MG exhibit the strongest transcriptomic disruption in STZ retinas, exceeding that of vascular endothelial cells and bipolar cells. Second, MG are not transcriptionally uniform: subclustering revealed four MG substates with distinct functional programs, and trajectory inference supported a structured progression in which early, high-potential MG states transition toward downstream reactive states. Third, STZ exposure was associated with reinforcement of inflammatory programs in multiple MG substates and with stronger expression of a Junb/Cebpb/Nfkbiz-centered co-expression network, suggesting that MG-driven stress-inflammatory signaling may represent an important axis of diabetic retinal remodeling. Together, these findings refine the current view of MG in diabetic retinopathy by emphasizing both their marked state heterogeneity and their potential role as active organizers of stress-associated retinal remodeling.

Our perturbation analysis placed MG at the top of the sensitivity hierarchy in STZ-induced DR, consistent with the conceptual role of MG as the principal homeostatic integrator of the neurovascular unit. Müller glia are strategically positioned to sense metabolic stress, including hyperglycemia and oxidative burden, maintain retinal homeostasis through neurotransmitter recycling and ion buffering, and communicate bidirectionally with the vascular compartment that governs the inner blood–retinal barrier (6, 7, 35, 36). The observation that vascular endothelial cells ranked second in perturbation further supports a coupled glia–vascular response rather than isolated changes in either compartment. Notably, prior work in the STZ model has largely emphasized the role of microglia and their activated subtypes, often positioning microglial alterations as a central event in early diabetic retinopathy (37). For instance, microglia–endothelial crosstalk has been reported to contribute to inflammatory signaling and vascular remodeling in DR, thereby reshaping the retinal inflammatory microenvironment and promoting angiogenesis (38). In contrast, we did not observe pronounced transcriptomic perturbation in microglia in our dataset, which may be attributable, at least in part, to the limited number of microglial cells captured (Ctrl, n = 27; STZ, n = 90). Together, these results support a model in which early diabetic stress triggers coordinated transcriptional reprogramming across the neurovascular unit, with MG functioning as a key amplifier that integrates neuronal distress signals, metabolic dysregulation, and paracrine inflammatory cues. From this perspective, MG may function not merely as passive responders, but as active amplifiers of diabetes-associated retinal stress signaling.

Although Müller glial heterogeneity has been reported in human and rat retinas with diabetic retinopathy (14), our study specifically focuses on dissecting Müller glial heterogeneity in a mouse model of DR. By re-integrating and subclustering MG, we resolved four substates (Clusters 0–3) that capture distinct biological themes. Cluster 1 exhibited an immediate-early/stress inflammatory signature characterized by AP-1 family factors and related response genes, consistent with a reactive state poised to orchestrate rapid transcriptional adaptation. Cluster 2 showed enrichment for lipid metabolism/transport and mitochondrial bioenergetics-related processes, suggesting a metabolically remodeled state potentially engaged in handling altered lipid flux and energy demands under diabetes. Cluster 3 displayed RNA-regulatory and extracellular matrix (ECM) features, which may reflect structural remodeling, reactive gliosis-associated matrix changes, or altered cell–cell/ECM interactions that accompany chronic stress. These observations support the idea that MG responses in diabetic retinopathy are not monolithic, but instead distributed across multiple coordinated substates with distinct functional emphases.

Notably, Cluster 0 showed a trend toward increased representation under STZ and was enriched for functions linked to phototransduction, neuronal support, vesicle trafficking, synaptic phagocytosis, and neurotransmitter-related transport. This pattern suggests that diabetic conditions may not only activate canonical reactive programs but may also be associated with MG substates displaying putative compensatory features, potentially related to neuronal support and damaged-material handling. In a degenerative microenvironment, increased MG phagocytic activity could represent an adaptive response that limits secondary inflammation by removing debris. However, persistent or excessive glial phagocytosis may also contribute to maladaptive synaptic remodeling, raising the possibility that Cluster 0 represents a state with both adaptive and potentially detrimental consequences, depending on the duration and intensity of diabetic stress.

CytoTRACE and pseudotime analyses converged on a coherent structure: Clusters 1 and 2 displayed relative transcriptional plasticity and occupied earlier positions on the inferred trajectory, whereas Cluster 0 localized toward a later region of the inferred trajectory. These analyses support the presence of state transitions and biased branch utilization in STZ retinas. This architecture supports the idea that MG heterogeneity is not merely a static mixture of subtypes but includes dynamic transitions among stress-adaptive states. Importantly, STZ conditions were associated with increased representation in the pooled dataset along a specific terminal branch (State 3), which was largely composed of Cluster 0. This biased branch utilization implies that diabetic stress does not uniformly activate MG; instead, it reshapes the MG state landscape by selectively strengthening particular transition routes. Importantly, these inferred trajectories should not be interpreted as evidence of developmental differentiation in mature MG, but rather as transitions among reactive transcriptional states under chronic metabolic stress.

The identification of two post-inflection fate directions provides additional nuance: one branch (cell fate 1) was dominated by photoreceptor-associated signatures, while the other branch (cell fate 2) was enriched for core stress/inflammatory genes. This bifurcation suggests that MG may diverge into alternative response modes—one oriented toward neuronal interaction/maintenance and the other toward inflammatory signaling. Such a framework helps reconcile the longstanding observation that MG responses in DR can be simultaneously protective and harmful, depending on the activated program and its persistence. Importantly, the CytoTRACE and pseudotime-based analyses in this study should not be interpreted as evidence of bona fide developmental differentiation in mature Müller glia. Rather, we interpret these ordered trajectories as reflecting relative transcriptomic plasticity and reactive state progression under chronic diabetic stress. In this context, the inferred substates may represent stress-adaptive or stress-maladaptive phenotypic programs that could be transient, reversible, or progressively stabilized depending on the duration and intensity of metabolic injury. Because the present dataset is cross-sectional, the reversibility, temporal ordering, and long-term stability of these inferred MG states cannot be determined here and will require future validation.

A striking feature of MG Cluster 0 was the elevated expression of genes typically associated with photoreceptors or bipolar cells, which was further supported by bubble-plot visualization under STZ conditions and reinforced by hdWGCNA Module 1 hub structure. There are multiple, non-mutually exclusive explanations for this phenomenon, and distinguishing among them is essential for mechanistic interpretation. One possibility is technical or compositional confounding, including ambient RNA contamination or doublets, which can introduce photoreceptor transcripts into non-photoreceptor clusters—particularly in retina datasets where photoreceptor RNA is abundant. A second possibility is biological acquisition of neuronal RNA through phagocytosis of damaged photoreceptor material; in this scenario, MG would contain photoreceptor-derived transcripts without necessarily adopting photoreceptor identity. A third, more speculative explanation is that diabetic stress induces partial derepression or atypical transcriptional programs in MG that resemble neuronal gene modules, perhaps reflecting altered chromatin accessibility or stress-induced transcription factor networks. Deng et al. identified a distinct Müller subpopulation in human and rat DR retinas characterized by high expression of the rod photoreceptor marker *Rho* (14). Using UMAP-based visualization and co-expression analyses, they showed that this subpopulation concomitantly expressed both photoreceptor-specific markers and canonical Müller glial markers. They interpreted this unusual co-expression pattern as indicative of a cell–cell interaction phenomenon, potentially reflecting the uptake of injured rod-derived material by Müller glia through phagocytosis. In our single-cell analysis of the STZ-induced DR mouse retina, we observed a highly similar feature, suggesting that this cross-lineage transcriptional signature may be conserved across species and may represent a shared stress-associated Müller glial response, whereby Müller cells internalize components of damaged rod photoreceptors. Phagocytic activation of Müller glia under retinal stress, including DR, has been proposed as a potentially adaptive response that may contribute to maintaining tissue integrity. For instance, in mouse models of retinal degeneration such as retinitis pigmentosa, Müller glia have been reported to serve as major phagocytes responsible for clearing dying rod photoreceptors, thereby supporting retinal homeostasis and limiting secondary damage that could accelerate disease progression (39). On this basis, we speculate that the Müller subpopulation exhibiting photoreceptor-associated transcripts in our dataset may reflect Müller cells that have acquired photoreceptor material via phagocytosis, although alternative explanations cannot be excluded. This pattern may represent a stress-associated adaptive mechanism and may be consistent with a compensatory response aimed at buffering photoreceptor injury under diabetic stress. Nevertheless, this interpretation remains provisional and will require spatially resolved validation, nucleus-based sequencing, and direct functional assays of MG phagocytic activity.

Our data are most consistent with a model in which Cluster 0 represents an STZ-enhanced MG state with increased neuron-interactive activity (e.g., debris clearance or synaptic remodeling). Nonetheless, rigorous validation is required. Spatially resolved assays (RNAscope/smFISH) could verify whether photoreceptor-associated transcripts localize within MG *in situ*. Nucleus-based sequencing can reduce cytoplasmic ambient RNA effects and help clarify whether these transcripts represent active transcription. In parallel, experimental assays of MG phagocytosis (e.g., uptake of labeled outer-segment material) under diabetic conditions could directly test the debris-clearance hypothesis.

Another key finding is that inflammatory activation was distributed across multiple MG substates, but was weighted most strongly toward Cluster 1. Functional enrichment analysis showed that inflammatory-response programs were activated across Clusters 1, 2, and 3, indicating that multiple MG substates participate in inflammatory remodeling under STZ. The intersection of inflammatory gene sets across these substates yielded nine shared genes (*Il33, Hmgb2, Nupr1, Zfp36, Cebpb, Ywhaz, Socs3, Camk2n1, Ier3*), suggesting a conserved “inflammation activation core” that spans distinct MG programs. Several of these genes are consistent with stress-alarm and transcriptional control systems: *Il33* is a well-known alarmin-like cytokine with immunomodulatory functions (40), *Hmgb2* and *Nupr1* are stress-associated inflammatory factors (41, 42), ZFP36 participates in post-transcriptional regulation of inflammatory mRNAs (43), and CEBPB is a central transcription factor that can coordinate inflammatory and injury-response programs (44). Prior literature further supports the biological plausibility of these prioritized genes. C/EBPβ has been implicated in inflammatory gene regulation in CNS glial cells, including the control of IL-1β-induced inflammatory programs in astrocytes and the promotion of pro-inflammatory microglial transcriptional states (45, 46). JUNB has likewise been identified as a key transcriptional modulator of macrophage activation, including regulation of inflammatory gene expression such as Il1b-associated responses (47). In contrast, SOCS3 appears to play context-dependent roles in retinal and neural inflammation. Together, these studies provide additional support for prioritizing *Cebpb*, *Junb*, and *Socs3* as biologically relevant regulators of inflammatory Müller glial states in diabetic retinopathy.

Although inflammatory activation was shared, Cluster 1 contained the largest complement of inflammation-associated genes and displayed strong enrichment for interferon signaling, IL-6 production, immune activation signatures, and T cell-related pathway terms. These results suggest that Cluster 1 may represent a prominent inflammatory MG state associated with inflammatory signaling in the diabetic retina. This observation further highlights the value of single-cell resolution for disentangling compartment-specific changes from global tissue-level averages. This substate-specific concentration of inflammatory features underscores the value of single-cell analysis, as such compartmentalized changes may be obscured in whole-retina measurements. In our study, hdWGCNA connects substate biology to co-expression architecture and highlights a Junb-centered inflammatory module. The hdWGCNA analysis adds a network-level view that complements differential expression and pathway enrichment. Two modules were particularly informative. Module 1 was preferentially expressed in Cluster 0 and featured hub genes and functional enrichments tied to visual processes, vesicle trafficking/endocytosis, ion transport, and oxidative stress, consistent with a neuron-interactive or compensatory response state. Module 6 was preferentially expressed in Cluster 1 and was dominated by immediate-early/stress response hubs (*Jun, Junb, Fos, Fosb, Btg2*), with functional enrichment for apoptosis, hypoxia response, angiogenesis-related programs, and immune/inflammatory signaling.

A key insight is that the Module 6 hub structure converged on AP-1 and related stress regulators, and PPI-based prioritization placed Junb at the top of the network. The observed increase in JUNB protein abundance in STZ retinas provides supportive tissue-level evidence that this transcriptional program is associated with biologically relevant signaling changes. Conceptually, this nominates AP-1/JUNB as a plausible candidate regulatory node linking diabetic stress to inflammatory transcriptional programs in MG. Whether JUNB is protective, maladaptive or context-dependent likely depends on timing, signal intensity, and interaction with other pathways (e.g., NF-κB, STAT signaling), and this is a concrete direction for future functional studies. Importantly, the co-expression modules identified here should be interpreted in the context of a single cross-sectional disease stage rather than as direct surrogates of longitudinal progression or phenotypic severity. Although modules such as Module 1 and Module 6 were preferentially activated under STZ conditions and were associated with distinct Müller glial substates, the present dataset does not include stage-resolved or longitudinal sampling across the diabetic course. Therefore, these modules are best viewed as STZ-associated transcriptional programs at the sampled time point, rather than as modules formally correlated with disease duration or progressive severity. Together with the increased CEBPB signal identified in the inflammatory context, these findings nominate Junb and Cebpb as candidate regulators for future mechanistic testing. Future studies incorporating earlier and later diabetic stages, or quantitative phenotypic readouts of retinal injury, will be required to determine how these network programs evolve over time and whether they track with worsening diabetic retinal pathology.

Together, our findings support a model in which STZ-induced diabetes is associated with multiple inferred MG substates displaying distinct transcriptional features: an AP-1–centered inflammatory state (Cluster 1/Module 6), metabolically remodeled states (Cluster 2), ECM/RNA-regulatory remodeling states (Cluster 3), and an expanded neuron-interactive/phagocytosis-associated late reactive state (Cluster 0/Module 1). More broadly, these Müller glial responses may be viewed within a conserved neurobiological framework in which local cellular remodeling contributes to larger compensatory and structural shaping programs across the CNS. In other neural contexts, dynamic reorganization of distributed circuits has been linked to preservation of functional outcomes after focal injury, supporting the idea that compensatory adaptation can emerge through coordinated structural and functional reweighting rather than simple restoration of the pre-injury state (48). Likewise, developmental and systems-level studies indicate that localized biological vulnerability can scale to broader neural signatures, in which regionally patterned structural adaptation is shaped by underlying cellular programs and is associated with later cognitive and psychiatric outcomes (49). In this context, the Müller glial programs identified here, including neuron-interactive/phagocytosis-associated responses, inflammatory remodeling, and structural support-related changes, may represent a retina-specific manifestation of a more general CNS principle: that glial and tissue-level compensation helps buffer local damage, reshape microenvironmental organization, and preserve neural integrity under persistent stress. This broader perspective may help explain why diabetic retinal remodeling includes both potentially protective and potentially maladaptive Müller glial states, depending on the balance between compensatory maintenance and chronic inflammatory reinforcement. This state architecture has practical implications. First, it argues that “reactive gliosis” in DR should be treated as a spectrum of discrete MG programs rather than a single uniform response. Second, it provides candidate markers and regulatory nodes (e.g., CEBPB, JUNB, Il33-associated modules) that may help refine disease-relevant MG biology beyond broadly expressed inflammatory transcripts. Third, it suggests that these observations may be relevant to future efforts aimed at modulating specific MG substates or transition routes, for example, dampening inflammatory programs while preserving potentially compensatory neuroprotective responses. More generally, the present study should be interpreted as a transcriptome-guided, hypothesis-generating analysis that prioritizes candidate molecules, modules, and state-associated programs for future functional and cell type-resolved validation.

However, we must also acknowledge that this study has several limitations. The STZ model primarily reflects insulin-deficient diabetes and may not capture all features of type 2 diabetes–associated DR; therefore, generalization across DR etiologies should be tested. The single-cell atlas reflects a particular sampling window and may not represent the full temporal evolution of MG state transitions. In addition, the public scRNA-seq dataset and our independent Western blot validation were not fully stage-matched. Therefore, the transcriptomic and protein-level findings should not be interpreted as strictly stage-matched validations of the same disease phase. Moreover, pseudotime inference suggests plausible trajectories but cannot establish lineage directionality without orthogonal evidence. Additionally, the photoreceptor-associated transcriptional features observed in Cluster 0 require *in situ* validation using multiplex immunofluorescence staining, and further investigation is needed to determine whether these signals reflect phagocytosed RNA or active intracellular transcription. Finally, although we observed a role for Müller glia in inflammatory responses and identified several inflammation-associated markers, these genes are unlikely to be Müller-glia specific, and the current whole-retina Western blot validation does not establish Müller cell-specific localization. Therefore, the identification of Müller glia–specific inflammatory markers will require additional validation, for example through co-localization analyses using double immunostaining or other cell-type–resolved approaches.

Future studies should integrate spatial transcriptomics or *in situ* validation to map MG substates within retinal layers and vascular proximity, and leverage MG-specific genetic perturbation to test causal roles of candidate regulators (e.g., conditional manipulation of *Junb* or *Cebpb* in MG). Functional assays assessing MG phagocytosis, synaptic remodeling, and barrier-supportive functions under diabetic stress would help connect transcriptomic states to physiological outcomes. Finally, the observed association of the lncRNA *Mir124-2hg* with the terminal branch suggests an additional layer of regulation worth exploring, potentially linking non-coding RNA control to MG state transitions.

## 5 Conclusion

In summary, our single-cell and network analyses suggest that MG are among the most transcriptionally perturbed retinal cell populations in the STZ-induced diabetic retina and exhibit pronounced heterogeneity organized into distinct substates and potential state-transition trajectories. STZ conditions were associated with a late reactive MG branch enriched for photoreceptor-associated and phagocytosis-related signatures, as well as with reinforcement of an AP-1/JUNB-centered inflammatory co-expression module in an inflammatory MG substate. These findings refine the cellular and molecular framework of MG involvement in diabetic retinal remodeling and nominate state-associated networks and regulators as candidate molecular hypotheses for future mechanistic and translational studies in diabetic retinopathy.

## Data Availability

The original contributions presented in the study are included in the article/**Supplementary Material**. Further inquiries can be directed to the corresponding author.
