# Supplementary material for: Single cell transcriptomic analysis reveals pathogenic cell heterogeneity and candidate inflammatory-associated markers in STZ-induced diabetic mouse retina
Source: Front Immunol. 2026 Apr 29;17:1827122. doi: 10.3389/fimmu.2026.1827122 (PMC13168181; doi:10.3389/fimmu.2026.1827122)
Supplement: Supplementary file 1 [file Table1.docx]

**Supplementary materials**

**
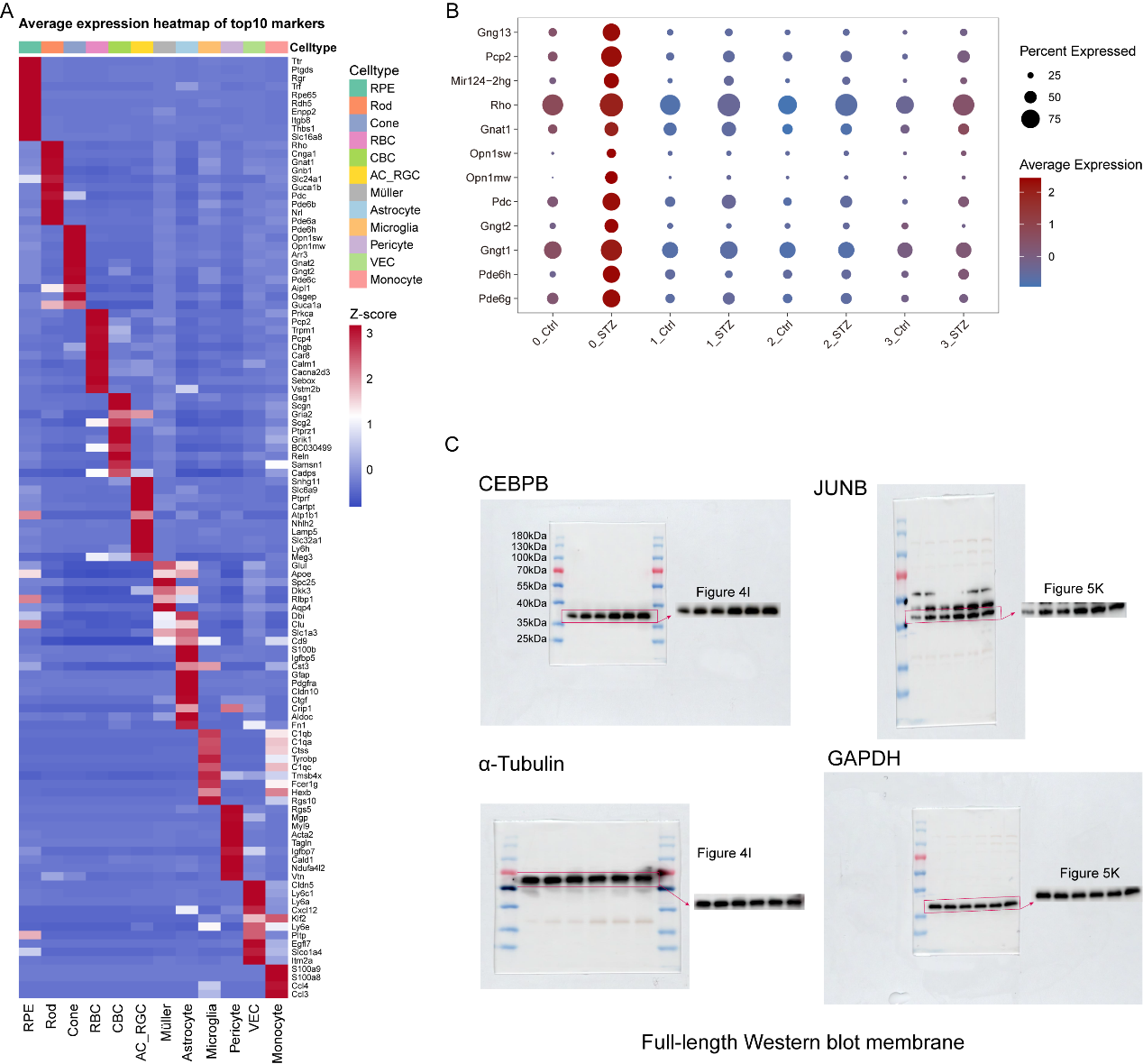
**

**Supplementary Figure S1**

Validation of retinal cell-type annotation, photoreceptor-associated gene expression across Müller glial substates, and full-length Western blot membranes. (A) Heatmap showing the average expression of the top 10 marker genes across annotated retinal cell types, including retinal pigment epithelium (RPE), rod photoreceptors (Rod), cone photoreceptors (Cone), rod bipolar cells (RBC), cone bipolar cells (CBC), amacrine cells/retinal ganglion cells (AC/RGC), Müller glia, astrocytes, microglia, pericytes, vascular endothelial cells (VEC), and monocytes. Color intensity represents row-scaled expression levels (Z score). These marker profiles support the accuracy of cell-type annotation in the single-cell dataset. (B) Dot plot showing the expression patterns of photoreceptor-associated genes across Müller glial subclusters (Clusters 0–3) under control (Ctrl) and STZ conditions. Dot size indicates the percentage of cells expressing each gene, and color intensity indicates average expression level. Genes shown include *Gng13*, *Pcp2*, *Mir124-2hg*, *Rho*, *Gnat1*, *Opn*1sw, *Opn1mw*, *Pde6b*, *Gngt2*, *Gngt1*, *Pde6h*, and *Pde6g*. The enrichment of these transcripts in Cluster 0, particularly under STZ conditions, further supports the presence of a photoreceptor-associated transcriptional program in this Müller glial substate. (C) Full-length Western blot membranes for CEBPB, JUNB, and the corresponding loading controls α-Tubulin and GAPDH.
